# Pharmacogenetics of Drug-Resistant Epilepsy (Review of Literature)

**DOI:** 10.3390/ijms222111696

**Published:** 2021-10-28

**Authors:** Beata Smolarz, Marianna Makowska, Hanna Romanowicz

**Affiliations:** 1 Laboratory of Cancer Genetics, Department of Pathology, Polish Mother’s Memorial Hospital Research Institute, Rzgowska 281/289, 93-338 Lodz, Poland; hanna-romanowicz@wp.pl; 2 Department of Anesthesiology and Intensive Care Medicine, Charité—Universitätsmedizin Berlin, Corporate Member of Freie Universität Berlin and Humboldt-Universität zu Berlin, Augustenburger Platz 1, 13353 Berlin, Germany; marianna.makowska@charite.de

**Keywords:** pharmacogenetics, drug-resistant epilepsy, genes, CYP2 family, MDR-1, GABA receptors, ion channels

## Abstract

Pharmacogenomic studies in epilepsy are justified by the high prevalence rate of this disease and the high cost of its treatment, frequent drug resistance, different response to the drug, the possibility of using reliable methods to assess the control of seizures and side effects of antiepileptic drugs. Candidate genes encode proteins involved in pharmacokinetic processes (drug transporters, metabolizing enzymes), pharmacodynamic processes (receptors, ion channels, enzymes, regulatory proteins, secondary messengers) and drug hypersensitivity (immune factors). This article provides an overview of the literature on the influence of genetic factors on treatment in epilepsy.

## 1. Introduction

The term pharmacogenetics, which Vogel [1] first used in 1959, refers to a department of clinical pharmacology that studies the effects of genetic factors on the action and fate of drugs in the body, with particular emphasis on inter-individual differences in drug response. 

The genetic basis of drug response is not a new issue. The first observations about the differentiated response to factors of external origin were made in ancient times by Pythagoras, who noticed that the consumption of broad beans caused potentially fatal complications in some people. It is now known that it is the genetically determined deficiency of glucose-6-phosphate dehydrogenase that is the cause of blood hemolysis after ingestion of broad beans (favism). During World War II, it was observed that hemolysis associated with malaria treatment was much more common in black American soldiers, which led to the distinction of a hereditary variant of glucose-6-phosphate dehydrogenase [2]. Reports of clinical significance about the different reaction of patients after the use of the same drug come from the late 1950s and concern the prolonged sagging effect of succinylcholine, conditioned by the occurrence of atypical butyrylcho-linesterase in serum [3,4].

Inter-individual genetic differences (genetic polymorphism), which are a consequence of mutations within the human genome, are usually the cause of observed differences in the enzymatic activity of proteins responsible for drug metabolism. In human DNA, there are often places that differ in individual people with a single nucleotide polymorphism (SNP). The density of SNP is estimated at 1 in 100–300 base pairs, for a total of 30 million SNPs across the human genome [5].

The population of epilepsy patients in Europe is approaching 6 million people, and the cost of treatment is EUR 18 billion per year [6,7]. The role of genetic factors, including genetic polymorphisms, and in particular their impact on clinical implications, is currently being studied extremely extensively in the context of drug-resistant epilepsy. In most patients, a single antiepileptic drug results in a satisfactory reduction in the number of seizures, or even their complete resolution. However, about 30% of patients have so-called drug-resistant epilepsy. 

According to the International League Against Epilepsy (ILAE) definition, epilepsy can be considered drug-resistant if the patient does not have seizure remission, despite the fact that he uses two appropriately selected, well-tolerated antiepileptic drugs (in appropriate doses, as monotherapy or in combination) [8].

In Poland, this problem affects about 100,000–120,000 patients. There is no single binding definition of drug-resistant epilepsy. In general, it can be concluded that it is characterized by the occurrence of seizures despite the use of optimal treatment. It is believed that the factors predisposing to the occurrence of this type of epilepsy are: the appearance of the disease before the age of 1 year, a high frequency of seizures before the start of treatment and structural changes in the brain, including malformations of the cerebral cortex [9]. 

Although potential risk factors for drug-resistant epilepsy are known, it has not yet been explained why in two patients with the same type of epilepsy or the same type of seizures, the effectiveness of treatment with antiepileptic drugs can be extremely different [10,11]. 

**Figure 1:**
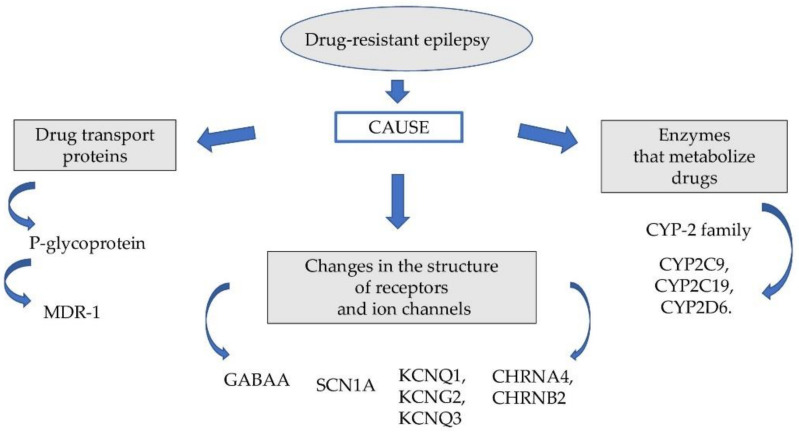
Factors affecting drug-resistant epilepsy.

The probable causes of this phenomenon are genetic factors responsible for changing the pharmacodynamic and pharmacokinetic properties of the drugs used. The group of these factors includes: genetically determined polymorphism of some microsomal enzymes (cytochrome P450 family 2 subfamily C member 9 (CYP2C9), cytochrome P450 family 2 subfamily C member 19 (CYP2C19)), P glycoproteins, multidrug resistance-associated protein (MRP) and disorders of pharmacodynamic function of neurotransmitter gamma-aminobutyric acid (GABA) receptors and ion channels (Figure 1).

## 2. Drug Transport Proteins

Glycoprotein-P (P-gp, MDR1) belongs to the superfamily membrane proteins ATP-binding cassette transporter (ABC). P-glycoprotein was first discovered in 1976 by Juliano and Ling [12]. P-glycoprotein is encoded by the multidrug resistance gene (MDR1). P-gp has a mass of 170 kDa and is a phosphorylate and glycosylated protein containing 1280 amino acids. It consists of two homologous halves, each containing six transmembrane domains (TMDs) and ATP nucleotide binding domain (NBD). P-glycoprotein acts as an adenosine triphosphate (ATP)-dependent pump, transporting substrates from the outer and inner layers of the cell membrane, leading to a decrease in their concentration inside the cell. Although P-gp was first described in tumour cells, where it caused resistance to anticancer drugs, its expression is also observed in healthy tissues. The presence of this protein was detected in the liver (epithelium of hepatocyte tubules and apical surfaces of bile ducts), enterocytes, renal cortex (blood–urine barrier), central nervous system (blood–brain barrier) [13], as well as the blood–testicle barrier [14] and placenta (mother–foetus barrier).

The tissue distribution of P-gp gives an important clue about its function. In most tissues, it is located on the apical surface of cells turned to the lumen of the bile ducts, intestinal lumen or vascular lumen. Such a location suggests that P-gp ejects its substrates into extracellular spaces. P-gp can therefore perform an important function as a natural protection of cells against toxic compounds. Intestinal P-gp reduces the absorption of toxins from food, while renal and hepatic P-gp mediates the elimination of these toxins in the urine and bile. In addition, the expression of P-gp in the blood–brain and blood–testis barriers reduces the passage of these substances to the brain and testicles. P-gp is capable of transporting corticosteroid hormones cortisol, corticosterone and aldosterone and presumably mediates the secretion of steroids from these tissues. Possible functions of P-gp are also i. a. lipid transport, effect on the level of cellular regulators and effect on apoptosis and cell differentiation [15]. Thus, this protein significantly affects the pharmacological properties of many drugs and their metabolites, modifying their bioavailability after oral administration, penetration into specific tissues and elimination from the body. 

Studies on P-gp expression in cell lines (Caco-2, L-MDR1) as well as in knock-out mice lacking the MDR1 gene have provided an opportunity to investigate the contribution of this protein to drug transport [16]. P-gp transports many structurally different hydrophobic and amphipathic drugs including a range of anti-cancer drugs, heart drugs, HIV protease inhibitors, beta-blockers and antihistamines. In rodents, three genes were distinguished: mdr1a, mdr1b and mdr1c. In mice, these genes form a cluster located on chromosome 5, while in rats it is located in the 4q11.12 region. Both protein products in rodents, mdr1a and mdr1b, perform a function analogous to human P-gp, while the mdr1c protein corresponds functionally to the human MDR3 transporter [16]. Mice lacking the gene for P-gp (knock-out mdra1) had 35 times higher concentrations of digoxin (P-gp substrate) in the brain and 2 times higher in plasma than control animals [17]. In addition, in the control group, 16% of the intravenous dose was excreted within the first 90 min. In comparison, only 2% of the administered dose was excreted by mice in the study group (knock-out mdra1) [18].

There is a lot of data [19,20,21,22] confirming that excessive activation of P-gp leads to the development of resistance to anticonvulsants drugs due to the excessive removal of these compounds through the blood–brain barrier back into the blood, resulting in too little penetration of these drugs into the brain. In addition, experimental studies indicate that administration of highly specific compounds that inhibit P-gp activity, such as tariquidar, increases the anticonvulsant effect of phenytoin [23] and even helps break resistance to phenobarbital [24]. In human medicine, one case of P-gp over-expression in the brain of a patient who died during an epileptic state has also been described [25]. A similar study was conducted in dogs, where a significant increase in P-gp expression in the capillary endothelium of the brain was also shown after spontaneous status epilepticus. The results indicate that prolonged seizures induce the appearance of P-gp over-expression in the brains of dogs, which may explain why delaying the start of treatment in the state of epilepsy leads to a decrease in the effectiveness of anticonvulsants, and may also be a mechanism contributing to the development of resistance to treatment in dogs with epilepsy, which affects about 20–40% of them [26].

Intraoperative studies using microdialysis indicate a different value of the concentration of antiepileptic drugs in the intercellular space, as well as their significantly lower concentration compared to serum [27], which suggests a variety of mechanisms involved in the transport of drugs and their distribution in the brain and makes this problem difficult.

MDR1 is over-expressed in neurons and astrocytes (in which MDR1 is normally not measurable) from lesions associated with active epileptogenic foci in human and rodent brain [28]. More than 10 times higher expression of the MDR1 gene in the epileptic focus of patients operated on for drug-resistant epilepsy compared to those in the control group was shown by Tishler et al. [29]. It was found that P-gp expression in endothelial cells was greater in people with drug-resistant epilepsy than among control group people with vascular malformations.

MDR1 polymorphism, which shapes the variable expression of P-gp, may therefore have an impact on the effectiveness of antiepileptic therapy. An increase in the expression of this gene leads to an increase in the amount of P-glycoprotein in endothelial cells of the blood–brain barrier and in astrocytes. The result of this process is a decrease in the parenchymal concentration of drugs despite the achieved therapeutic level in the blood.

The MDR1 gene, also known as the ABCB1 gene, encoding P-gp is located on chromosome 7 (7q21.1) and contains 28 exons. It is highly polymorphic. Unlike mutations, genetic polymorphism is responsible for subtle changes in DNA and is easily passed on to subsequent generations. Single nucleotide polymorphism affects both the coding and non-coding fragments of DNA and can lead to a change in the amino acid of the encoded protein or the level of its expression, and consequently to a change in its structure or function.

Half of the SNPs identified so far in MDR1 are located in the coding part of the gene, and one of them, located in exon 26, seems to have functional significance. The allele frequency for most SNPs in the coding regions is low (8% in ethnically different populations). The exceptions are three single nucleotide polymorphisms: in exon 12 (rs1128503, 1236C>T), in exon 21 (rs2032582, 2677G>T/A) and exon 26 (rs1045642, 3435C>T). The biological significance of alleles of the above polymorphisms and their haplotypes is being intensively studied in the context of drug-resistant epilepsy [30]. It has been proven that these polymorphisms are in conjugate equilibrium and several of their haplotypes are related to phenotypes and protein overexpression [31,32,33,34]. 

Studies of the relationship between MDR1 polymorphisms and response to antiepileptic treatment conducted in ethnically different populations have so far yielded contradictory results. It should be emphasized that most of the data from the world literature concern the polymorphism of single nucleotides MDR1 3435C>T (rs1045642). In patients with drug-resistant epilepsy in studies by van Vliet et al. [35] and Lazarowski et al. [36], C3435T/CC genotype is associated with an increase in P-gp expression, which affects the concentration of antiepileptic drugs in plasma. 

In the field of pharmacogenomic studies of membrane drug transporters, the association of drug resistance in epilepsy with the genotype 3435CC of the MDR1 gene [37,38] has been described, but other researchers have not confirmed the existence of such a relationship [39,40,41,42,43,44]. Based on the results obtained by Alpman et al. [45] it was concluded that MDR1 C3435T and G2677AT polymorphisms are not associated with multidrug resistance, but CC3435/GG2677 compound genotype may affect the effectiveness of treatment [45]. In the Polish population, statistically significant associations were found between C3435T and G2677T/A polymorphisms and the occurrence of drug-resistant epilepsy [46,47].

Inter-individual differences in the effectiveness of antiepileptic drugs make treating epilepsy a challenge. In the work of Zhao et al. [48], the effect of polymorphisms in the gene encoding P glycoprotein, ATP Binding Cassette Subfamily B Member 11 (C1236T, G2677T/A and C3435T) on levetiracetam disposition in Chinese Uygur children with epilepsy was evaluated. A total of 245 Uygur children with epilepsy (drug-resistant, *n* = 117 (men:women = 53:64) and drug responsive, *n* = 128 (men:women = 76:52)) were examined. The incidence of the genotypes, alleles, haplotypes or diplotypes ABCB1 C1236T, G2677T/A and C3435T did not differ significantly between the two groups. Significantly higher levetiracetam concentrations and serum concentrations/body weight dose were observed in ABCB1 genotypes 2677-GT, TT, GA and AT and 3435-TT carriers compared with GG and CC carriers. ABCB1 G2677T/A and C3435T may affect levetiracetam disposition and therapeutic efficacy in Uygur children with epilepsy. Genetic analysis can be a valuable tool for predicting responses to antiepileptic drugs before starting treatment and may contribute to the creation of personalized medicine for Uygur children with epilepsy [48].

In the work of Gao et al. [49] genotypes of loci rs1922242, rs2235048, rs10808072, rs868755 and rs1202184 of the MDR1 gene were analysed in a group of 200 children with epilepsy and 100 healthy children from the control group [49]. There were no significant differences in the distribution of genotypes and alleles loci rs1922242, rs2235048, rs10808072 and rs868755 between drug-resistant and drug-sensitive groups. For locus rs1202184, a significant difference in genotypic distribution was found. There was no significant difference in the incidence of different haplotypes between the two groups. Genotypes of the locus rs1202184 of the MDR1 gene are associated with treatment-resistant epilepsy in children in whom the AA genotype plays a dominant role. The results of in vitro and in vivo studies suggest that the variability in MDR1 transporter expression may be closely related to drug resistance. Although there is strong support for this hypothesis from molecular studies, population studies of ABCB1 polymorphisms diverge in their conclusions and additional studies are needed [50].

The relationship between MDR1 C3435T gene polymorphism and refractory epilepsy in childhood remains controversial. Lv et al. [51] searched the PubMed, Medline, Embase and CNKI databases for studies published up to October 2016 that assessed the relationship between MDR1 C3435T polymorphism and drug-resistant epilepsy in children. Eleven studies were included in the systematic review and meta-analysis involving 863 cases in the drug-resistant epilepsy group and 915 cases in the drug-resistant epilepsy group. The analysis showed that there was no significant association of MDR1 C3435T polymorphism with the overall risk of drug resistance. However, the allelic association of MDR1 C3435T and the association of MDR1 3435 CC genotype with the risk of drug resistance were significant in the European population and the “>2010” group based on subgroup analysis in the year of publication. The relationship between MDR1 C3435T polymorphism and drug-resistant epilepsy in children requires further validation [51].

Tamimi et al. showed a strong association between ABCB1 gene polymorphisms rs2235048, rs1045642, rs2032582 and rs1128503 and antiepileptic resistance among women but not men [52]. The strongest relevant associations were for haplotypes containing T-2677G_1236 in the two SNP haplotypes three -SNPs-haplotypes and four -SNPs-haplotypes. The data suggest that there is a gender-dependent relationship between ABCB1 genetic polymorphisms and the response to antiepileptic drugs. Additionally, ABCB1 haplotypes affect the response to antiepileptic drugs. More research is needed to confirm the results of this study. ATP-binding cassette superfamily G member 2 (ABCG2) is another ABC transporter that also plays a key role in multidrug resistance to chemotherapeutic agents [53,54]. 

A growing body of evidence indicates that genetic polymorphisms of members of the ATP-binding cassette superfamily, such as ABCC2 and ABCG2, alter responses to antiepileptic drugs (AEDs); however, this evidence is controversial and inconclusive [55,56,57,58]. To provide strong evidence for the relationship between common polymorphisms in ABCC2 and ABCG2 responses and AED in epilepsy patients, meta-analysis studies were conducted [59]. ABCG2, located on chromosome 4q22.1, contains several functional genetic polymorphisms, such as rs2231137 and rs2231142. A significant association of ABCC2 rs717620 polymorphism with antiepileptic drug resistance was found in the general combined population. Using a recessive model, a similarly significant association of ABCC2 rs3740066 with antiepileptic drug resistance was observed in the general combined population. However, ABCC2 rs2273697, ABCG2 rs2231137 and rs2231142 were not associated with reactivity on AED. Research suggests that ABCC2 rs717620 and rs3740066 are risk factors that predict a response to AED in patients with epilepsy.

The cited data from the literature indicate that there is no clear answer to the question regarding the role of the polymorphisms of MDR1/ABCB1 gene in drug-resistant epilepsy. However, the results of the above studies may be caused by a more complex relationship between polymorphisms than previously thought. It should be noted that P-gp and multidrug resistance-associated protein (MRP) are not the only transporters taken into account in the conditions of drug resistance.

Awasthi et al. [60] showed that the glycoprotein RLIP76/RALBP1 also plays an important role in the transport of antiepileptic drugs, which is also involved in the process of limiting the influx of these drugs into the nervous tissue and leads to resistance to treatment. However, these observations were not confirmed by subsequent studies [61,62].

Bearing in mind the therapeutic prospects, it is impossible to overlook the fact that in an experiment using the rat model of epilepsy, the administration of phenytoin with the P-gp inhibitor—tariquidar—resulted in a reduction in the frequency of seizures [22]. Perhaps this will indicate the direction of further attempts to be made in relation to patients with drug-resistant epilepsy.

## 3. Enzymes That Metabolize Drugs

It is well known that the mechanisms of drug metabolism affect their concentration in the blood, the route of excretion, toxicity and their availability at the site of action. Most drugs are metabolized by the cytochrome P450 family of enzymes. Among the 18 cytochromes P450 (CYP) families identified, the most diverse is the cytochrome P450 family 2 (CYP2), responsible for the first phase of the metabolism of exogenous molecules. It includes many enzymes encoded by polymorphic genes. The most important in the context of clinical consequences are CYP2C9, CYP2C19 and CYP2D6. A variable incidence of CYP genetic variants was observed, which is responsible for the specific activity of drug metabolizing enzymes within populations inhabiting different geographical regions of the world [63].

Biotransformation and elimination of drugs, including antiepileptic drugs, takes place by oxidation reactions catalysed by various cytochrome P450 enzymes and glucuronidation, for the course of which two are important—CYP2C9 and CYP2C19. Their genetic polymorphism affects the rate of drug metabolism, leading to different sensitivity to their dose, severe idiosyncrasy reactions and even toxic symptoms. Data on the relationship between genetic background and antiepileptic drugs metabolism are mainly derived from clinical observations of often small groups of patients in whom clinical monitoring of changes is short-lived. This presents a significant difficulty in assessing the impact of genetic differences on the biotransformation of antiepileptic drugs [64]. 

Despite these limitations, the effect of CYP2C9 and CYP2C19 polymorphism on phenytoin metabolism was noted. So far, 13 alleles of the CYP2C9 gene have been identified, of which CYP2C9*2 and CYP2C9*3 resulted from mutations in the CYP29C*1 coding sequence leading to the substitution of one amino acid—R144C and I359L, respectively. This is associated with reduced activity of the enzyme to metabolize phenytoin [65]. 

Research by van der Weide et al. [66] involving 60 patients treated with chronic phenytoin who have identified at least one of the CYP2C9*2 or CYP2C9*3 alleles, shows that the dose of CYP2C9*3 necessary to achieve the therapeutic serum concentration was 37% lower compared to the dose in patients with CYP2C9*1. Similar results were obtained by Watanabe et al. for different CYP2C19 alleles in the Japanese population [67].

In the study by Lopez-Garcia et al. [68], CYP2D6, CYP2C9, CYP2C19 and CYP3A4 polymorphisms were identified in a rigorously selected population of paediatric patients with drug-resistant epilepsy. The corresponding SNPs with pharmacogenomic associations were CYP2D6*2 (rs16947) reduced activity and CYP2D6*4 (rs1065852), CYP2C19*2 (rs4244285) and CYP3A4*1B (rs2740574) by association with poor metabolism. The strongest risk factors were found in the AA genotype and the SNP allele rs3892097 from the CYP2D6 gene, followed by the A and T SNP alleles rs2740574 and rs2687116, respectively, from CYP3A4. The most significant coexistence occurred between homozygous AA genotype rs3892097 and AA genotype rs2740574 with 78.3% in patients with drug-resistant epilepsy compared with 14.3% in control patients. The results demonstrated an important role of the allelic variant CYP 3A4*1B as a risk factor for the development of drug resistance. SNPs and CYP2D6, CYP2C19 haplotypes may affect the response to antiepileptic drugs [68].

Makowska et al. [69] evaluated the relationship between the polymorphisms rs1799853 (430C>T) and rs1057910 (1075A>C) of the CYP2C9 gene and the polymorphism of rs4244285 (c.681G>A) of the CYP2C19 gene, and the incidence of drug-resistant epilepsy in children. The CT genotype of the rs1799853 CYP2C9 polymorphism and the GA genotype of the rs4244285 polymorphism of the CYP2C19 gene were shown to result in an increased risk of epilepsy. It has also been shown that the occurrence of C-G-A haplotype in relation to the polymorphism rs1799853 of the CYP2C9 gene and the polymorphism rs4244285 of the CYP2C19 gene may be associated with a reduced risk of epilepsy. In the case of rs1799853 polymorphism in the CYP2C9 gene, the presence of the T allele quadrupled the risk of drug resistance in patients diagnosed with epilepsy. The results obtained indicate that the polymorphisms rs1799853 and rs1057910 CYP2C9 and the polymorphism rs4244285 CYP2C19 may be associated with the occurrence of drug-resistant epilepsy in children [69].

The aim of the work of the team of Emich-Widera et al. [70] was an assessment of the relationship between CYP3A5 polymorphism and MDR1 C3435T polymorphism with focal drug-resistant epilepsy in children and adolescents up to 18 years of age. The study included 85 patients aged 33 months to 18 years, suffering from epilepsy, partly responding well to treatment, partly drug-resistant. The study did not confirm the association between CYP3A5*3 polymorphism and C3435T polymorphism in the MDR1 gene and pharmacological epilepsy. The results of the study do not confirm the prognostic value of the studied polymorphisms in the prognosis of drug resistance in epilepsy.

## 4. Changes in the Structure of Receptors and Ion Channels

Maintaining a balance between inhibition and synaptic stimulation is essential to maintain normal levels of neuronal excitability. This balance is provided by the organization of the neural network. Its main elements are stimulation of glutamatergic basal cells and inhibition of GABAergic interneurons. GABA works through three types of membrane receptors, of which γ-aminobutyric acid type A (GABAA) is widely represented in the central nervous system. The GABAA receptor is a pentameric complex. This complex consists of two subunits α and two subunits β (GABA binding site and one subunit of the γ or δ). Activation of GABAA causes hyperpolarization of neurons, which prevents the formation and spread of convulsive activity [71]. Mutations in the GABA receptor subunit, especially GABAA, have been shown to be associated with genetically determined seizures and epileptic syndromes. Literature data indicate that the presence of a mutant α2 subunit has been associated with the development of autosomal dominant myoclonal epilepsy of adolescence [72]. The polymorphism G 1465A of the GABAB1 receptor has been shown to be associated with susceptibility to temporal epilepsy and affects the severity of the disease [73]. However, these results were not supported by subsequent studies by other authors [74,75].

Ion channels: potassium [76,77], sodium [76,77,78], chloride and calcium [76,77] participate in the process of epileptogenesis (Table 1). At the root of seizures are mutations in the genes encoding ligand-dependent or voltage-gated ion channel subunits.

Neuronal nicotinic receptors (nAChRs) form a heterogeneous family of ion channels that are differently expressed in many regions of the central nervous system (CNS) and peripheral nervous system. Sleep-related hypermotor epilepsy (SHE), previously known as nocturnal frontal lobe epilepsy [79], is focal epilepsy characterized by seizures with either complex hyperkinetic mechanisms, an asymmetric tonic/dystonic form, or both, occurring primarily during sleep [80]. A familial form of SHE with autosomal dominant inheritance (ADSHE) has been described [81,82]. SHE is the first epilepsy syndrome in which genetic etiology has been documented [83]. It is also the first described channelopathy of epilepsy, as it was initially associated with mutations in genes encoding subunits of the neural nicotinic acetylcholine receptors CHRNA4, CHRNB2 and CHRNA2 [84,85,86,87,88,89,90,91,92,93]. SHE then associated with mutations in several other genes encoding proteins involved in various biological pathways, such as CRH [94], KCNT1 [83,95,96,97,98,99,100,101,102], DEPDC5 [103,104,105,106,107,108,109,110], NPRL2, NPRL3 [111,112] and PRIMA1 [113]. A new missense mutation has been found in the CABP4 gene encoding the neuronal binding protein Ca2+4 (CaBP4), including 11 individuals diagnosed with ADSHE a (p.G155D) in the Ca2+4 binding protein (CABP4) in a Chinese family with autosomal dominant nocturnal frontal lobe epilepsy [114].

The discovery of new drugs and the development of anticonvulsive pharmacotherapy is largely possible thanks to the use of animal models in preclinical studies. It is important that all parameters are carefully controlled, including the choice of species, strains, age and sex of animals [115]. The ideal preclinical model of epileptogenesis should exhibit many characteristics associated with epilepsy in humans, including either seizure-induced nerve cell death, subsequent cognitive impairment, other co-occurring behaviours, or in combination [116].

The maximal electroshock seizure (MES) test and pentylenotetrazol (PTZ) injections were developed 60 years ago and are still relevant and most frequently chosen in testing new antiepileptic drugs [117]. Caic acid (KA) was one of the first compounds used to model temporal lobe epilepsy (TLE) in rodents. Ka-induced epilepsy in rodents is thought to resemble human temporal epilepsy to a large extent, depending on the route of administration. Intracerebral KA injection has been shown to most accurately mimic human TLE, while systemic administration of KA causes greater pathological damage, both in the brain and on the periphery [118]. One of the most commonly used models of epileptic seizures of the temporal lobe is kindling. It consists of the gradual development of electrographic and behavioural seizures with repeated stimulation of animals by pro-convulsive chemicals such as PTZ [119]. Despite the painstaking nature of the procedure, kindling gives the opportunity to study the dynamics of epileptogenic processes, which are particularly relevant for TLE. Kindling has become one of the most important models of epilepsy used to study neurochemical and long-term structural changes in the brain [120]. The search for seizure mechanisms can provide insight into general brain and consciousness function, and animal models of epilepsy will continue to promote advances in both the study of epilepsy and neurophysiological processes [121].

Antiepileptic drugs that block sodium channels lead to the predominance of arousal processes and worsen the condition of patients with this syndrome. The possibility of pharmacological activation of potassium channels from the KV7 family on the extinction of seizures has led to experimental observations on the action of retigabine. It has been shown to be active in blocking partial seizures, also resistant to treatment. Retigabine has so far proven to be the only anticonvulsive that directly activates the opening of KCNQ (KV7) channels [122]. 

The drug was registered in the European Union in March 2011. Its level of efficacy and safety was established in the RESTORE 1 and 2 clinical trials, which were conducted in 17 countries and included 843 drug-resistant patients who had previously taken one to three antiepileptic drugs. In these studies, a reduction in seizure rates of 50 percent or more was observed in significantly more patients with partial seizures who were given retigabine in addition to their antiepileptic drugs than in patients who received placebo [123,124]. 

Ineffectiveness and difficulties during antiepileptic treatment can also result from unexpected, sometimes dangerous, side effects of drugs. It has been documented that in the Asian population, the occurrence of the HLA-B*1502 allele is associated with an increased risk of Stevens–Johnson syndrome (SJS) after carbamazepine or phenytoin [125].

These observations have not been confirmed in the Caucasian population related to carbamazepine [126]. 

In patients with the HLA-B*1502 allele, antiepileptic drugs with an aromatic ring in their structure, i.e., carbamazepine, oxcarbazepine, lamotrigine and phenytoin, have been shown to cause Stevens–Johnson syndrome (SJS) or toxic epidermal necrolysis (Lyell’s syndrome, LS). Other alleles that increase the risk of SJS and LS in patients receiving phenytoin are HLA-B*1301, Cw*0801 and DRB1*1602 [127].

Carbamazepine (CBZ) is one of most used antiepileptic drugs. However, CBZ resistance is common in patients with epilepsy, and genetic polymorphisms can affect the response to antiepileptic drugs. The relationship between the polymorphisms rs3812718 and rs2298771 of the SCN1A gene and the risk of CBZ resistance in epilepsy remains controversial. To more accurately assess the linked association, a meta-analysis was conducted to investigate the contribution of two single nucleotide polymorphisms of SCN1A that may give resistance to CBZ. The PubMed, Embase and Web of Science databases were searched for eligible studies. All controlled clinical trials on the association of SCN1A, rs3812718 and rs2298771 polymorphisms with CBZ resistance in epilepsy were included. A significant association was found between rs2298771 (GG vs. GA + AA) and CBZ resistance in patients of Asian origin with epilepsy. No association was observed between rs3812718 polymorphism and CBZ reactivity. The results indicate that patients of Asian descent with epilepsy and SCN1A polymorphism rs2298771, especially with the GG genotype, may be at risk of CBZ resistance [128].

The metabolism and efficacy of carbamazepine may be affected by xenobiotic receptors, especially the pregnane X receptor (PXR), the constitutive androstane receptor (CAR) and the aryl hydrocarbon receptor (AHR). An interaction was observed between the CAR variant rs2502815 and the CBZ response: in patients based on CBZ monotherapy and combination therapy, the GG genotype of the CAR variant rs2502815 (as compared with wild-type homozygous) was independently associated with the CBZ response. The results of haplotype analysis and gene interaction in CBZ response were negative. This study is the first to indicate a potentially significant interaction between CAR rs2502815 polymorphism and CBZ response in patients with epilepsy [129].

The cited research results confirm the influence of genetic factors on the occurrence of immune processes causing life-threatening reactions to antiepileptic drugs. This fact is somewhat reflected in clinical practice—the treatment process in a thirty-year-old patient with skin and organ lesions that occurred after the use of lamotrigine, successfully used immunoglobulin G at a dose of 1 mg/kg for 5 days [130].

## 5. Currently Used Antiepileptic Drugs

Over the past two decades, a group of 23 drugs for the treatment of epileptic seizures has been approved by the U.S. Food and Drug Administration (FDA) [131,132,133,134,135,136,137,138,139,140].

**Figure 2:**
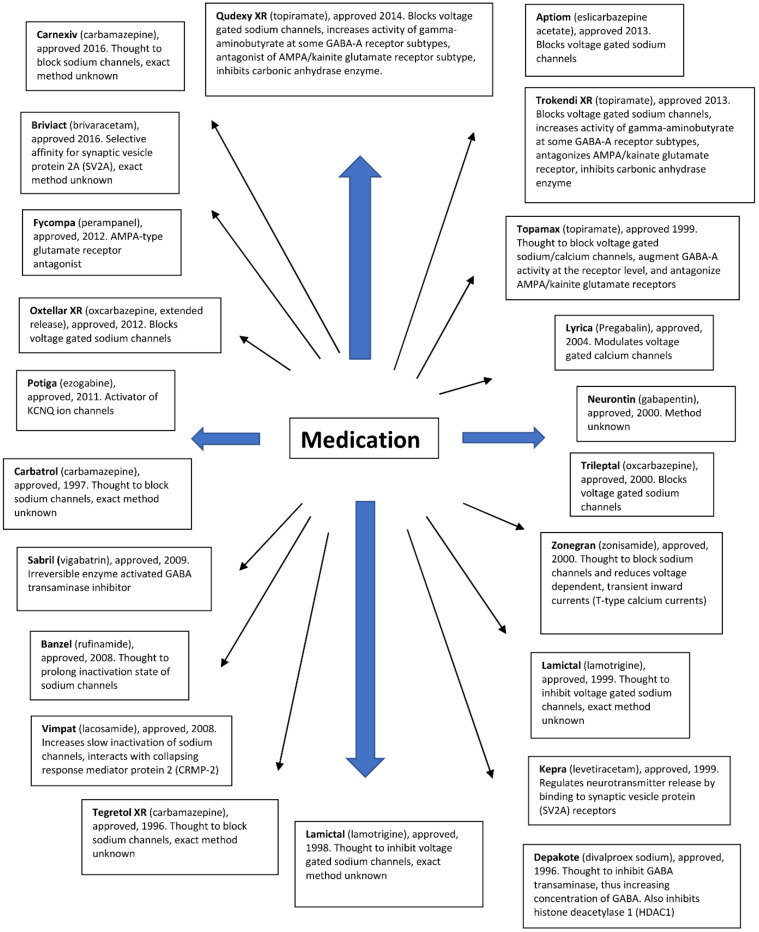
Antiepileptic drugs and their mode of action.

Antiepileptic drugs show different modes of action. They aim to reduce neuronal excitability, which can be achieved by modulating the activity of several relevant target drugs. In the literature, resistance has been described for a large number of antiepileptic drugs with different mechanisms of action (Figure 2). Many drugs inhibit voltage-gated sodium channels. Some antiepileptic drugs have secondary mechanisms of action, thereby increasing the activity of GABAA receptors. Other antiepileptic drugs mainly affect GABA synthesis or GABAA receptors without affecting sodium channel function. Another group of drugs targets the 2A synaptic vesicle glycoprotein (SV2A). This allows you to modulate the release of neurotransmitters to synapses. Several approved LPPs act on other receptors or have poorly understood modes of action.

Despite such a wide range of drugs with different methods of action, each category of currently approved drugs may become ineffective in many patients due to the acquisition of drug resistance. Because resistance to multiple drugs often develops simultaneously, it is likely that broad-spectrum resistance has little to do with the different ways in which individual drugs work. 

Eslicarbazepine acetate is a third-generation antiepileptic drug, which, when taken orally, is rapidly and intensively converted into eslicarbazepine. The reduction in the frequency of seizures in patients treated with eslicarbazepine is only partial in most patients, and many of them suffer from significant drug resistance, which requires a change in treatment [141]. P-glycoprotein, encoded by the ABCB1 gene, is expressed throughout the body and may affect the pharmacokinetics of several drugs. The effects of three common ABCB1 polymorphisms (i.e., C3435T or rs1045642, G2677A or rs2032582 and C1236T or rs1128503) on the pharmacokinetics and safety of eslicarbazepine were investigated. No significant association was observed between the sex, race and polymorphism of ABCB1 and the pharmacokinetic variability of eslicarbazepine. In contrast, the ABCB1 C1236T C/C diplotype was significantly associated with the occurrence of drug resistance. This is the first study published to date to evaluate the pharmacogenetics of eslicarbazepine. More research is needed on large samples to compare the results obtained [142].

Anti-seizure medications (ASM) prevent epileptic seizures occurring. Genetic markers of resistance to individual ASMs can help clinicians make more informed choices for their patients. It will perform a genome-wide association study (GWAS) on individuals responding to specific ASMs or functionally related groups of ASMs, using non-responders as controls. It will also identify several potential regions of interest, but this will not detect relevant genome-wide loci for the ASM-specific response [143]. GWAS studies did not show a predictive value of common genetic variants for ASM response status. The identified suggestive loci will require replication in future larger-scale studies.

## 6. Conclusive Summary

The ineffectiveness of the therapy used and the occurrence of side effects of drugs is a clinical problem of every doctor, regardless of specialization. It is particularly important in the case of epilepsy, due to the fact that it affects as many as 30% of patients who do not respond to treatment. The role of genetic factors in the etiopathogenesis of drug resistance in epilepsy seems indisputable. The dynamic development of new research methods, as well as the use of molecular biology methods in medicine, enable more and more accurate diagnostics, aimed at determining the genetic basis of the disease in some cases. An opportunity for clinicians is also the possibility of using pharmacogenetics. Confirmation of the relationship between P-gp and drug resistance would allow the development of new treatment directions, for example, combination therapy of an antiepileptic drug with a P-gp inhibitor, or the selection of risk groups for developing drug-resistant epilepsy in the future, which could facilitate the decision to modify treatment faster or to have surgery earlier. It is to be hoped that the wider use of genetic methods in both diagnosis and therapy is becoming a matter of time, and their use will contribute to reducing the extent of the problem of drug-resistant epilepsy. 

## Figures and Tables

**Table 1:** Ion channels involved in the pathogenesis of idiopathic epilepsy.

Voltage-Gated Ion Channels	Chromosome (Locus)	Gene	Gene Product	Syndrome
Potassium channel	20q13	KCNQ2	potassium channel KCNQ2	mild familial seizures of newborns
	8q24	KCNQ3	potassium channel KCNQ3	
Sodium channel	2q22–23	SCN2A	sodium channel subunits α2	benign familial neonatal-infantile seizures (BFNIS)
	19q13	SCN1B	regulatory subunit β of sodium channel	generalized epilepsy with febrile seizures (GEFS+1)
	2q24	SCN1A	sodium channel subunits α1	generalized epilepsy with febrile seizures (GEFS+2), Dravet syndrome (severe myoclonic epilepsy in infancy, SMEI)
	2q22–23	SCN2A	sodium channel subunits α2	generalized epilepsy with febrile seizures (GEFS+4)
Chloride channel	3q27–28	CLNC2	chloride channels CLC-2	child absence epilepsy (CAE) juvenile absence epilepsy (JAE) juvenile myoclonic epilepsy (JME)
Calcium channel	6p12	EFHC1	regulator of voltage-dependent ion channels	JME
	16p13	CACNA1H	T-type calcium channel	CAE
	2q22–23	CACNB4	subunit β2 of calcium channel	JME

## Data Availability

Data sharing is not applicable to this article.
